# 1-Benzyl­sulfanyl-2-[(2-chloro­phen­yl)diazen­yl]benzene

**DOI:** 10.1107/S1600536810025730

**Published:** 2010-07-07

**Authors:** Pranjit Barman, Tirtha Bhattacharjee, Rupam Sarma

**Affiliations:** a Department of Chemistry, National Institute of Technology, Silchar 788 010, Assam, India; b Department of Chemistry, Indian Institute of Technology, Guwahati 781 039, Assam, India

## Abstract

The title compound, C_19_H_15_ClN_2_S, a divalent organosulfur compound belonging to the class of *ortho*-mercaptoazo compounds, is non-ionic in nature. The azo group in the mol­ecule is moved away from the S atom to attain the stable *trans*-azo configuration. Here the S atom is not electron deficient, so no intra­molecular N⋯S inter­action exists. Due to steric reasons, the mol­ecule is non-planar: the chlorophenyl and benzyl rings are oriented at dihedral angles of 3.21 (8) and 78.18 (5)°, respectively, with respect to the thiophenyl ring. There are no hydrogen bonds and the crystal structure is stabilized by van der Waals inter­actions.

## Related literature

For background to our study of the effect of substituents at the 2′- and 4′- positions of azobenzene-2-sulfenyl compounds and related structures, see: Karmakar *et al.* (2001 ▶); Sanjib *et al.* (2004 ▶); Kakati & Chaudhuri (1968 ▶). For the reactivity of sulfenyl compounds towards biomolecules, see: Fontana *et al.* (1968 ▶).
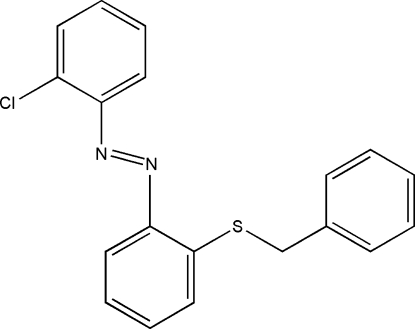


## Experimental

### 

#### Crystal data

C_19_H_15_ClN_2_S
*M* *_r_* = 338.85
Monoclinic, 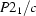

*a* = 15.493 (2) Å
*b* = 5.4218 (8) Å
*c* = 20.206 (3) Å
β = 96.055 (9)°
*V* = 1687.8 (4) Å^3^
*Z* = 4
Mo *K*α radiation
μ = 0.35 mm^−1^
*T* = 296 K
0.21 × 0.16 × 0.14 mm

#### Data collection

Bruker APEXII CCD area-detector diffractometer
16728 measured reflections
3139 independent reflections
2140 reflections with *I* > 2σ(*I*)
*R*_int_ = 0.042

#### Refinement

*R*[*F* ^2^ > 2σ(*F* ^2^)] = 0.037
*wR*(*F* ^2^) = 0.088
*S* = 1.01
3139 reflections
208 parameters
H-atom parameters constrained
Δρ_max_ = 0.14 e Å^−3^
Δρ_min_ = −0.17 e Å^−3^

### 

Data collection: *APEX2* (Bruker, 2001 ▶); cell refinement: *SAINT* (Bruker, 2001 ▶); data reduction: *SAINT*; program(s) used to solve structure: *SHELXS97* (Sheldrick, 2008 ▶); program(s) used to refine structure: *SHELXL97* (Sheldrick, 2008 ▶); molecular graphics: *SHELXTL* (Sheldrick, 2008 ▶); software used to prepare material for publication: *SHELXTL*.

## Supplementary Material

Crystal structure: contains datablocks I, global. DOI: 10.1107/S1600536810025730/rk2215sup1.cif

Structure factors: contains datablocks I. DOI: 10.1107/S1600536810025730/rk2215Isup2.hkl

Additional supplementary materials:  crystallographic information; 3D view; checkCIF report

## Figures and Tables

**Fig. 1.**
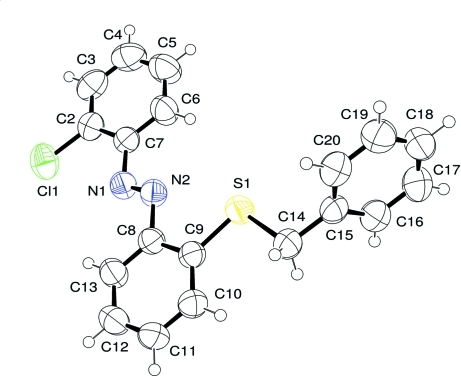
The molecular structure of the title compound, showing the atom-labelling scheme. Displacement ellipsoids are drawn at the 50% probability level. H atoms are presented as a small spheres of arbitrary radius.

**Fig. 2.**
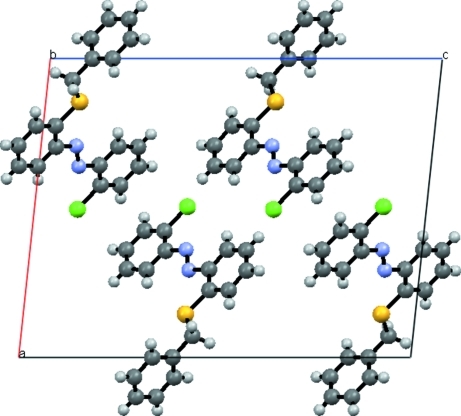
The packing diagram of the title compound, viewed along the b axis.

## supplementary crystallographic information

### Comment

To investigate the effect of substituents on the 2'- and 4'-positions of azobenzene-2-sulfenyl compounds (Karmakar *et al.*, 2001; Sanjib  *et al.* 2004) in the formation of thiadiazolium structures by *ortho* azo-sulfur interaction and to study the reactivity of sulfenyl compounds towards biomolecules (Fontana *et al.*, 1968), the title compound (Fig. 1) is studied. The sulfenyl sulfur S1 is *sp*^3^ hybridized and nucleophilic in nature for which the azo group moves away from it to attain the stable *trans*-azo-configuration. Such a situation was also found in azobenzene-2-sulfenyl cyanide (Kakati & Chaudhuri, 1968). The C*sp*^3^–Ssp^3^ [1.8064 (19)Å] bond is a normal covalent bond. The C*sp*^2^–S [1.7655 (19)Å] bond length is in the expected range and N1═N2 [1.247 (2)Å] bond length is in the expected range of an azo N═N bond length so there will be no resonance donating electron delocalization from the sulfenyl sulfur S1 into the extended conjugated system of the *trans*-azobenzene unit [no d-resonance between (vacant d orbital) S1 and the aromatic π-cloud] and no sulfur-*ortho*-azo interaction. The benzyl unit is moved away from the thiophenyl unit due to steric reason. There are no hydrogen bonds and the crystal structure is stabilized by Van der Waal's interactions (Fig.2).

### Experimental

To a solution of 2-benzylthioaniline in glacial acetic acid an equimolar amount of 2-chloronitrosobenzene in glacial acetic acid was added and stirred for 45 minutes. During stirring temperature was maintained between 323 to 343 K. Then the solution was kept in a dark place overnight at room temperature. Orange crystals of 2'-chloro-2-thiobenzylazobenzene were obtained, filtered off, washed with dilute acetic acid and dried, which melted at 414 K.

### Refinement

Hydrogen atoms were placed in calculated positions with C–H = 0.93Å and 0.97Å for aromatic and methylene H respectively and refined as riding with *U*_iso_(H) = 1.2*U*_eq_(C).

### Crystal data

**Table:**

C_19_H_15_ClN_2_S	*F*(000) = 704
*M**_r_* = 338.85	*D*_x_ = 1.334 Mg m^−^^3^
Monoclinic, *P*2_1_/*c*	Melting point: 414 K
Hall symbol: -P 2ybc	Mo *K*α radiation, λ = 0.71073 Å
*a* = 15.493 (2) Å	Cell parameters from 3629 reflections
*b* = 5.4218 (8) Å	θ = 4.0–25.5°
*c* = 20.206 (3) Å	µ = 0.35 mm^−^^1^
β = 96.055 (9)°	*T* = 296 K
*V* = 1687.8 (4) Å^3^	Needle, orange
*Z* = 4	0.21 × 0.16 × 0.14 mm

### Data collection

**Table:**

Bruker APEXII CCD area-detector diffractometer	2140 reflections with *I* > 2σ(*I*)
Radiation source: fine-focus sealed tube	*R*_int_ = 0.042
graphite	θ_max_ = 25.5°, θ_min_ = 2.0°
φ– and ω–scans	*h* = −18→18
16728 measured reflections	*k* = −5→6
3139 independent reflections	*l* = −24→24

### Refinement

**Table:**

Refinement on *F*^2^	Primary atom site location: structure-invariant direct methods
Least-squares matrix: full	Secondary atom site location: difference Fourier map
*R*[*F*^2^ > 2σ(*F*^2^)] = 0.037	Hydrogen site location: inferred from neighbouring sites
*wR*(*F*^2^) = 0.088	H-atom parameters constrained
*S* = 1.01	*w* = 1/[σ^2^(*F*_o_^2^) + (0.0254*P*)^2^ + 0.3762*P*] where *P* = (*F*_o_^2^ + 2*F*_c_^2^)/3
3139 reflections	(Δ/σ)_max_ < 0.001
208 parameters	Δρ_max_ = 0.14 e Å^−^^3^
0 restraints	Δρ_min_ = −0.16 e Å^−^^3^

### Special details

**Table:**

Geometry. All s.u.'s (except the s.u. in the dihedral angle between two l.s. planes) are estimated using the full covariance matrix. The cell s.u.'s are taken into account individually in the estimation of s.u.'s in distances, angles and torsion angles; correlations between s.u.'s in cell parameters are only used when they are defined by crystal symmetry. An approximate (isotropic) treatment of cell s.u.'s is used for estimating s.u.'s involving l.s. planes.
Refinement. Refinement of *F*^2^ against ALL reflections. The weighted *R*-factor *wR* and goodness of fit *S* are based on *F*^2^, conventional *R*-factors *R* are based on *F*, with *F* set to zero for negative *F*^2^. The threshold expression of *F*^2^ > σ(*F*^2^) is used only for calculating *R*-factors(gt) *etc*. and is not relevant to the choice of reflections for refinement. *R*-factors based on *F*^2^ are statistically about twice as large as those based on *F*, and *R*-factors based on ALL data will be even larger.

### Fractional atomic coordinates and isotropic or equivalent isotropic displacement parameters (Å^2^)

**Table:**

	*x*	*y*	*z*	*U*_iso_*/*U*_eq_	
S1	0.14479 (3)	−0.10089 (10)	0.08652 (2)	0.06781 (18)	
Cl1	0.50431 (4)	0.68379 (13)	0.11015 (3)	0.0911 (2)	
N2	0.29240 (10)	0.2094 (3)	0.08968 (7)	0.0599 (4)	
N1	0.36050 (10)	0.3311 (3)	0.10180 (7)	0.0619 (4)	
C7	0.35709 (12)	0.5064 (4)	0.15401 (8)	0.0567 (5)	
C2	0.42276 (12)	0.6809 (4)	0.16243 (9)	0.0619 (5)	
C3	0.42403 (16)	0.8556 (4)	0.21239 (11)	0.0794 (6)	
H3	0.4678	0.9737	0.2174	0.095*	
C6	0.29365 (14)	0.5079 (4)	0.19748 (10)	0.0759 (6)	
H6	0.2493	0.3917	0.1927	0.091*	
C14	0.07222 (13)	−0.3539 (4)	0.06272 (10)	0.0729 (6)	
H14A	0.0480	−0.3352	0.0167	0.087*	
H14B	0.1036	−0.5089	0.0670	0.087*	
C5	0.29587 (17)	0.6806 (5)	0.24774 (11)	0.0908 (7)	
H5	0.2534	0.6793	0.2770	0.109*	
C4	0.36019 (18)	0.8533 (5)	0.25459 (11)	0.0900 (7)	
H4	0.3608	0.9707	0.2882	0.108*	
C9	0.22512 (12)	−0.1349 (3)	0.03137 (8)	0.0555 (5)	
C8	0.29495 (12)	0.0306 (3)	0.03821 (8)	0.0557 (5)	
C13	0.36052 (13)	0.0163 (4)	−0.00329 (9)	0.0700 (6)	
H13	0.4061	0.1286	0.0015	0.084*	
C10	0.22486 (13)	−0.3157 (4)	−0.01772 (9)	0.0657 (5)	
H10	0.1796	−0.4289	−0.0232	0.079*	
C11	0.29086 (14)	−0.3283 (4)	−0.05799 (10)	0.0726 (6)	
H11	0.2897	−0.4508	−0.0903	0.087*	
C12	0.35836 (15)	−0.1636 (4)	−0.05150 (10)	0.0776 (6)	
H12	0.4023	−0.1734	−0.0794	0.093*	
C16	0.00046 (14)	−0.5197 (4)	0.15911 (10)	0.0756 (6)	
H16	0.0450	−0.6346	0.1663	0.091*	
C15	0.00057 (13)	−0.3535 (4)	0.10759 (9)	0.0611 (5)	
C20	−0.06639 (15)	−0.1870 (4)	0.09826 (11)	0.0791 (6)	
H20	−0.0673	−0.0738	0.0636	0.095*	
C19	−0.13194 (15)	−0.1838 (5)	0.13896 (13)	0.0887 (7)	
H19	−0.1767	−0.0695	0.1317	0.106*	
C18	−0.13148 (16)	−0.3489 (5)	0.19034 (12)	0.0871 (7)	
H18	−0.1756	−0.3469	0.2182	0.104*	
C17	−0.06553 (16)	−0.5167 (5)	0.20023 (11)	0.0887 (7)	
H17	−0.0650	−0.6298	0.2349	0.106*	

### Atomic displacement parameters (Å^2^)

**Table:**

	*U*^11^	*U*^22^	*U*^33^	*U*^12^	*U*^13^	*U*^23^
S1	0.0700 (3)	0.0685 (4)	0.0670 (3)	−0.0132 (3)	0.0166 (2)	−0.0146 (3)
Cl1	0.0710 (4)	0.1094 (5)	0.0936 (4)	−0.0207 (3)	0.0127 (3)	0.0060 (4)
N2	0.0640 (10)	0.0614 (10)	0.0542 (9)	−0.0060 (9)	0.0061 (7)	−0.0025 (8)
N1	0.0625 (10)	0.0661 (11)	0.0570 (9)	−0.0087 (9)	0.0061 (8)	−0.0041 (8)
C7	0.0628 (12)	0.0573 (12)	0.0491 (10)	−0.0007 (10)	0.0015 (9)	0.0007 (9)
C2	0.0628 (12)	0.0643 (13)	0.0565 (11)	−0.0009 (10)	−0.0035 (9)	0.0068 (10)
C3	0.0925 (17)	0.0642 (15)	0.0764 (15)	−0.0095 (12)	−0.0156 (13)	−0.0024 (12)
C6	0.0813 (15)	0.0829 (16)	0.0654 (13)	−0.0117 (13)	0.0172 (11)	−0.0086 (12)
C14	0.0833 (15)	0.0647 (14)	0.0733 (13)	−0.0175 (11)	0.0208 (11)	−0.0102 (11)
C5	0.1033 (19)	0.102 (2)	0.0701 (15)	0.0007 (16)	0.0217 (13)	−0.0170 (14)
C4	0.117 (2)	0.0813 (18)	0.0691 (15)	0.0060 (16)	−0.0025 (15)	−0.0192 (13)
C9	0.0639 (12)	0.0526 (12)	0.0497 (10)	0.0006 (9)	0.0048 (9)	0.0017 (9)
C8	0.0619 (12)	0.0567 (12)	0.0479 (10)	0.0004 (10)	0.0038 (9)	−0.0004 (9)
C13	0.0678 (13)	0.0787 (15)	0.0651 (12)	−0.0100 (11)	0.0147 (10)	−0.0071 (11)
C10	0.0750 (14)	0.0611 (13)	0.0613 (12)	−0.0067 (11)	0.0081 (10)	−0.0063 (10)
C11	0.0916 (16)	0.0682 (15)	0.0598 (12)	0.0007 (13)	0.0162 (11)	−0.0110 (10)
C12	0.0816 (16)	0.0851 (17)	0.0697 (13)	−0.0026 (13)	0.0243 (11)	−0.0105 (12)
C16	0.0816 (15)	0.0670 (15)	0.0794 (14)	0.0016 (12)	0.0139 (12)	0.0072 (12)
C15	0.0672 (13)	0.0541 (13)	0.0622 (12)	−0.0105 (10)	0.0078 (10)	−0.0069 (10)
C20	0.0867 (16)	0.0689 (15)	0.0822 (15)	−0.0033 (13)	0.0122 (13)	0.0096 (12)
C19	0.0774 (16)	0.0795 (17)	0.1111 (19)	0.0054 (13)	0.0188 (14)	−0.0010 (16)
C18	0.0842 (17)	0.0862 (19)	0.0961 (18)	−0.0160 (15)	0.0348 (14)	−0.0174 (15)
C17	0.108 (2)	0.0824 (18)	0.0803 (15)	−0.0092 (16)	0.0300 (14)	0.0124 (13)

### Geometric parameters (Å, °)

**Table:**

S1—C9	1.7655 (19)	C9—C8	1.401 (2)
S1—C14	1.8064 (19)	C8—C13	1.386 (2)
Cl1—C2	1.730 (2)	C13—C12	1.377 (3)
N2—N1	1.247 (2)	C13—H13	0.9300
N2—C8	1.425 (2)	C10—C11	1.374 (3)
N1—C7	1.425 (2)	C10—H10	0.9300
C7—C2	1.387 (3)	C11—C12	1.371 (3)
C7—C6	1.385 (2)	C11—H11	0.9300
C2—C3	1.383 (3)	C12—H12	0.9300
C3—C4	1.372 (3)	C16—C15	1.377 (3)
C3—H3	0.9300	C16—C17	1.384 (3)
C6—C5	1.379 (3)	C16—H16	0.9300
C6—H6	0.9300	C15—C20	1.373 (3)
C14—C15	1.506 (2)	C20—C19	1.373 (3)
C14—H14A	0.9700	C20—H20	0.9300
C14—H14B	0.9700	C19—C18	1.371 (3)
C5—C4	1.364 (3)	C19—H19	0.9300
C5—H5	0.9300	C18—C17	1.367 (3)
C4—H4	0.9300	C18—H18	0.9300
C9—C10	1.394 (2)	C17—H17	0.9300
			
C9—S1—C14	102.30 (9)	C9—C8—N2	115.27 (16)
N1—N2—C8	114.54 (15)	C12—C13—C8	120.21 (19)
N2—N1—C7	113.69 (15)	C12—C13—H13	119.9
C2—C7—C6	118.60 (18)	C8—C13—H13	119.9
C2—C7—N1	117.50 (17)	C11—C10—C9	120.64 (19)
C6—C7—N1	123.87 (18)	C11—C10—H10	119.7
C3—C2—C7	120.70 (19)	C9—C10—H10	119.7
C3—C2—Cl1	118.97 (17)	C12—C11—C10	121.27 (19)
C7—C2—Cl1	120.33 (16)	C12—C11—H11	119.4
C4—C3—C2	119.4 (2)	C10—C11—H11	119.4
C4—C3—H3	120.3	C11—C12—C13	119.37 (19)
C2—C3—H3	120.3	C11—C12—H12	120.3
C5—C6—C7	120.4 (2)	C13—C12—H12	120.3
C5—C6—H6	119.8	C15—C16—C17	120.4 (2)
C7—C6—H6	119.8	C15—C16—H16	119.8
C15—C14—S1	108.44 (13)	C17—C16—H16	119.8
C15—C14—H14A	110.0	C20—C15—C16	118.22 (19)
S1—C14—H14A	110.0	C20—C15—C14	120.84 (19)
C15—C14—H14B	110.0	C16—C15—C14	120.9 (2)
S1—C14—H14B	110.0	C15—C20—C19	121.5 (2)
H14A—C14—H14B	108.4	C15—C20—H20	119.3
C4—C5—C6	120.1 (2)	C19—C20—H20	119.3
C4—C5—H5	119.9	C18—C19—C20	120.0 (2)
C6—C5—H5	119.9	C18—C19—H19	120.0
C5—C4—C3	120.7 (2)	C20—C19—H19	120.0
C5—C4—H4	119.7	C17—C18—C19	119.3 (2)
C3—C4—H4	119.7	C17—C18—H18	120.3
C10—C9—C8	117.69 (17)	C19—C18—H18	120.3
C10—C9—S1	124.96 (15)	C18—C17—C16	120.5 (2)
C8—C9—S1	117.34 (14)	C18—C17—H17	119.7
C13—C8—C9	120.82 (17)	C16—C17—H17	119.7
C13—C8—N2	123.91 (17)		
			
C8—N2—N1—C7	−179.16 (14)	S1—C9—C8—N2	−0.1 (2)
N2—N1—C7—C2	−168.03 (16)	N1—N2—C8—C13	−10.4 (3)
N2—N1—C7—C6	13.7 (3)	N1—N2—C8—C9	169.87 (16)
C6—C7—C2—C3	−1.2 (3)	C9—C8—C13—C12	−0.8 (3)
N1—C7—C2—C3	−179.52 (16)	N2—C8—C13—C12	179.50 (18)
C6—C7—C2—Cl1	179.09 (15)	C8—C9—C10—C11	−0.5 (3)
N1—C7—C2—Cl1	0.8 (2)	S1—C9—C10—C11	−179.50 (15)
C7—C2—C3—C4	1.0 (3)	C9—C10—C11—C12	−0.4 (3)
Cl1—C2—C3—C4	−179.29 (17)	C10—C11—C12—C13	0.6 (3)
C2—C7—C6—C5	0.3 (3)	C8—C13—C12—C11	0.0 (3)
N1—C7—C6—C5	178.57 (19)	C17—C16—C15—C20	−0.2 (3)
C9—S1—C14—C15	178.30 (14)	C17—C16—C15—C14	179.67 (19)
C7—C6—C5—C4	0.7 (3)	S1—C14—C15—C20	76.9 (2)
C6—C5—C4—C3	−0.9 (4)	S1—C14—C15—C16	−103.02 (19)
C2—C3—C4—C5	0.1 (3)	C16—C15—C20—C19	0.2 (3)
C14—S1—C9—C10	1.43 (19)	C14—C15—C20—C19	−179.71 (19)
C14—S1—C9—C8	−177.62 (14)	C15—C20—C19—C18	0.1 (4)
C10—C9—C8—C13	1.0 (3)	C20—C19—C18—C17	−0.4 (4)
S1—C9—C8—C13	−179.84 (15)	C19—C18—C17—C16	0.3 (4)
C10—C9—C8—N2	−179.24 (16)	C15—C16—C17—C18	0.0 (3)
